# Towards sustainable demersal fisheries: NepCon image acquisition system for automatic *Nephrops norvegicus* detection

**DOI:** 10.1371/journal.pone.0252824

**Published:** 2021-06-16

**Authors:** Maria Sokolova, Fletcher Thompson, Patrizio Mariani, Ludvig Ahm Krag

**Affiliations:** 1 Section for Fisheries Technology, National Institute of Aquatic Resources, Technical University of Denmark, Hirtshals, Denmark; 2 Section for Oceans and Arctic, National Institute of Aquatic Resources, Technical University of Denmark, Lyngby, Denmark; Vellore Institute of Technology: VIT University, INDIA

## Abstract

Underwater video monitoring systems are being widely used in fisheries to investigate fish behavior in relation to fishing gear and fishing gear performance during fishing. Such systems can be useful to evaluate the catch composition as well. In demersal trawl fisheries, however, their applicability can be challenged by low light conditions, mobilized sediment and scattering in murky waters. In this study, we introduce a novel observation system (called NepCon) which aims at reducing current limitations by combining an optimized image acquisition setup and tailored image analyses software. The NepCon system includes a high-contrast background to enhance the visibility of the target objects, a compact camera and an artificial light source. The image analysis software includes a machine learning algorithm which is evaluated here to test automatic detection and count of Norway lobster (*Nephrops norvegicus)*. NepCon is specifically designed for applications in demersal trawls and this first phase aims at increasing the accuracy of *N*. *norvegicus* detection at the data acquisition level. To find the best contrasting background for the purpose we compared the output of four image segmentation methods applied to static images of *N*. *norvegicus* fixed in front of four test background colors. The background color with the best performance was then used to evaluate computer vision and deep learning approaches for automatic detection, tracking and counting of *N*. *norvegicus* in the videos. In this initial phase we tested the system in an experimental setting to understand the feasibility of the system for future implementation in real demersal fishing conditions. The *N*. *norvegicus* directed trawl fishery typically has no assistance from underwater observation technology and therefore are largely conducted blindly. The demonstrated perception system achieves 76% accuracy (F-score) in automatic detection and count of *N*. *norvegicus*, which provides a significant elevation of the current benchmark.

## Introduction

Crustacean trawl fisheries are very important socially, gastronomically and economically worldwide [1]. The Norway lobster (*Nephrops norvegicus*) (hereafter referred to as *N*. *norvegicus*) fishery is one of the most commercially important in the northeastern Atlantic region. Approximately 95% of *N*. *norvegicus* catches in Europe are caught using demersal trawls [2] and this fishery is challenged by several unique conditions. Firstly, *N*. *norvegicus* are benthic animals and inhabit muddy sediments in which they make burrows [3]. Thus, this species can be caught only when they are out of burrows on the seabed [4]. There are currently no robust indicators for fishers to identify when *N*. *norvegicus* are outside burrows and available to trawling. For this reason, demersal trawl fishery targeting *N*. *norvegicus* conducts trawl tows based on experience only with no real-time assessment of the fishing operations efficiency. Fishing operations can last for several hours and may often result in catches with little or none of the target species, but with catches of unintended species and sizes [5]. It is indeed a mixed fishery because of the high presence of co-habiting fish species and the small mesh sizes needed to retain *N*. *norvegicus* efficiently [6, 7]. This challenges the sustainability of this fishery and can have severe ecological and economic consequences which, in some jurisdictions, may restrict fishing opportunities. For example, in EU waters where fisheries activities are quota regulated and subject to the landing obligation [8].

The *N*. *norvegicus* fishery would benefit expressively from improved decision support tools in terms of real-time monitoring and description of the ongoing catching process and this is the main motivation of this study. Since *N*. *norvegicus* live in close proximity to the seabed, acoustic detection is difficult [9]. Similarly, optical detection methods are challenged in demersal trawl fisheries by the dark murky conditions at the seabed [10]. Video monitoring of catches on commercial vessels are mostly performed on deck for control purposes when the catch has been already extracted from the fishing gear. But it has been shown that the survival rates of some commercially important bycatch species that are lifted on board and then released are decreased [7]. Therefore, the development of an underwater monitoring tool can provide valuable decision support during the single fishing operations, helping to reduce bycatches and fuel consumption due to reduced fishing time [11]. Although promising results have been already obtained by integrating novel imaging systems into the fishing gear [11–13], the method is not yet fully developed, due to the difficulties in collecting underwater images of sufficient quality for species recognition.

Different systems for underwater image acquisition have been developed and used in fishing gear technologies, for example to observe fish behavior, verify the fish species observed on the echograms and to monitor the gear dynamics [10, 13, 14]. However, these applications are challenged by poor underwater visibility conditions by ground gear sediment mobilization especially in low-headline demersal trawls. High-resolution acoustic methods [9, 15] and range-gated systems [16] may improve observation of fish in natural conditions, but specific applications in catch monitoring are not available yet for the demersal trawl fishery.

Fully integrated vision-based monitoring tools in demersal fisheries could be achieved by using existing widely used portable cameras and the image analysis can be automatized with the aid of software packages that are supported by a large open source community. These developments include fast, minimal or no parameter object segmentation methods for image and video data, creating a fruitful ground for of *in-situ* catch count monitoring. The NepCon system described in this study is the first step towards a cost effective, fully in-trawl integrated system. This first development phase is concerned with the identification of materials and contrasting colors optimizing image segmentation of *N*. *norvegicus*, which is a critical step for the future automated detection and counting components of the NepCon system in trawls.

Due to the known challenges of image data collection from demersal trawls, we are interested in developing an enclosed section in the aft part of the trawl for making optical observations. The section will be made with an opaque PVC-coated tarpaulin material covering the netting inside the trawl to present a static observation environment and be flexible and robust enough to withstand commercial application. The background will enable optical catch monitoring during fishing operation and further automatization of the video analysis in terms of *N*. *norvegicus* recognition and counting. Under such static conditions we have an opportunity to implement classic computer vision approaches for object segmentation that utilize features such as edges, color and texture. In this study we focus on the selection of the background, lighting and camera conditions, as well as exploring classic and deep learning computer vision object segmentation methods, so that future at sea trawl application of the system will be well-adapted to the challenge.

Classic image processing and object segmentation from the background provide satisfactory results for identifying animals in the images based on the pixel intensities [17]. This method does not require high computational power and is easy to implement in comparison with training a deep learning-based object segmentation, meaning a fast and low-cost solution for the fishery. This method is based on pre-defined parameters, and will identify any “object” that satisfies the predetermined criteria [18]. In other words, this method is not semantic and is dependent on the input image or video quality and predefined parameters. However, it shows to be efficient and does not require the extensive labeled training data.

State-of-the-art deep learning algorithms are shown to have high accuracy in object segmentation and classification and have outperformed classic computer vision approaches in these tasks [19]. However, to benefit from the deep learning approach one needs to provide a large, annotated dataset and powerful hardware to train the model [20]. Most of the deep learning models are trained on extensive datasets containing millions of images of common objects that can be found in everyday life. The models are robust enough that the learned segmentation knowledge gained in one problem can be transferred, solving another specific task within the related field—the technique commonly called transfer learning [21]. In this study we explored the ability of a deep learning model trained on an extensive dataset to segment *N*. *norvegicus* without modifying the layers and weights of the network through specific training. We have selected mask R-CNN (mask region based convolutional neural network) that in addition to the bounding box and class identification outputs a pixel-wise mask of an object [22]. The mask also gives an opportunity to convert the pixel length of the object to an estimate of the actual size of an object [23–25].

To automatically detect *N*. *norvegicus* in the videos we apply an image processing pipeline and algorithm for identifying *N*. *norvegicus*-like objects in images, and then track these objects between frames in videos. The automatic count is compared with the ground truth of manually counted *N*. *norvegicus*.

The observation conditions during fishing can change dramatically depending on the daytime and depth as well as the fishing grounds. Where possible, we use automatic settings on the camera, such as white balancing and exposure, to reflect the fact that the actual lighting conditions in the trawl are unknown and subject to change, which means the camera must adapt to the conditions. Furthermore, the end users (i.e. fishers) require an automated solution with minimal background knowledge in photography or computer vision to operate.

We conclude this paper with evaluation of the proposed underwater image acquisition system and recommendations for further development of the in-trawl observation scene for image data collection for the automated detection in demersal trawls and challenges with the in-trawl implementation. Further improvement of the object detection and classification with the application of deep-learning approaches is dependent on the increase of the underwater images database.

## Methods and materials

### Ethical statement

We used 16 individuals of live *N*. *norvegicus* in the experiment. The individuals that were fixed in front of the background for images acquisition and used for the first set of videos were killed prior to the experiment through transection of the cephalothorax. *N*. *norvegicus* used for collecting videos of group of individuals were alive and the experiment was done in fresh seawater supplied by the seawater intake at the North Sea Science Park, Hirtshals, Denmark. *N*. *norvegicus* is not an endangered or protected species and no permit is required to conduct the experiment on invertebrates.

### Replication of the fishing conditions in experiment

**Fig 1:**
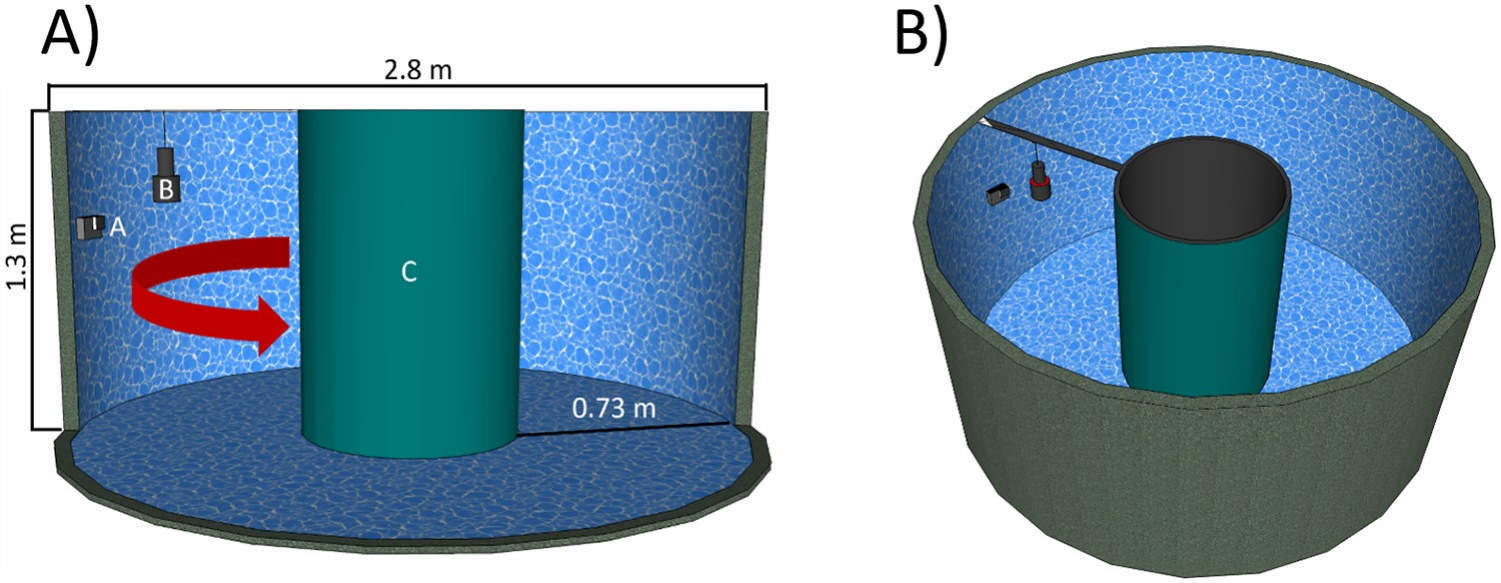
Experiment setup. A) Dimensions of the experimental round tank (front wall removed) with GoPro camera (A) attached to the inside tank wall via holder and Velcro-tape; the light (INON) (B); inner cylinder covered by the tarpaulin sheet (C); arrow indicates the water flow direction; B) Experimental tank: view from top.

We performed the experiment in a custom-made round tank facility made to simulate conditions during trawling at the Technical University of Denmark (DTU Aqua) (Fig 1). A current of approximately 2 knots was created using an industrial centrifugal pump (Tapflo^®^) to approach the towing speed during demersal *N*. *norvegicus* directed fishing [26].

The test backgrounds were four sheets of PVC-coated tarpaulin with the colors: yellow, green, orange and white. They were fixed on the center cylinder in pairs: yellow/green and orange/white, each color covering half of the circumference.

The background colors choice was based on the literature review [27] of the visibility of different colors under water: in clear fresh water, murky fresh water, sea water, turbid sea water. According to the review orange is the most visible color in turbid waters, therefore was chosen as a test color. Yellow is the second bright color in terms of underwater visibility, white is the color that reflects all the wavelengths of the visible spectrum and the green is a complementary color to the red target species [18, 28].

After the images and video footage were recorded for the first piece of tarpaulin, it was replaced by another color set.

We used a GoPro Hero 7 camera for images and videos acquisition. The camera was attached to the inner wall of the tank with Velcro^®^ tape and GoPro housing facing the inner cylinder covered by tarpaulin. After finalizing data collection for a background color, the camera was moved to the opposite inner wall of the tank for the second background color recording. To approximate low-light fishing conditions we isolated the facility from outside illumination.

As a light source we used INON^®^ diver underwater light (LF1300-EWf) with maximum intensity of 1300 lumens and 100 degree underwater coverage. We fixed INON^®^ underwater light above *N*. *norvegicus* for the observation scene. The light was pointing downwards facing the tank bottom to minimize the presence of shadows on the background from the *N*. *norvegicus* passing in front of the camera (Fig 1).

We moved the light source vertically closer to and further away from the camera (± 10 cm) and object to optimize appropriate illumination conditions of the target *N*. *norvegicus* and background.

### Data sampling

**Fig 2:**
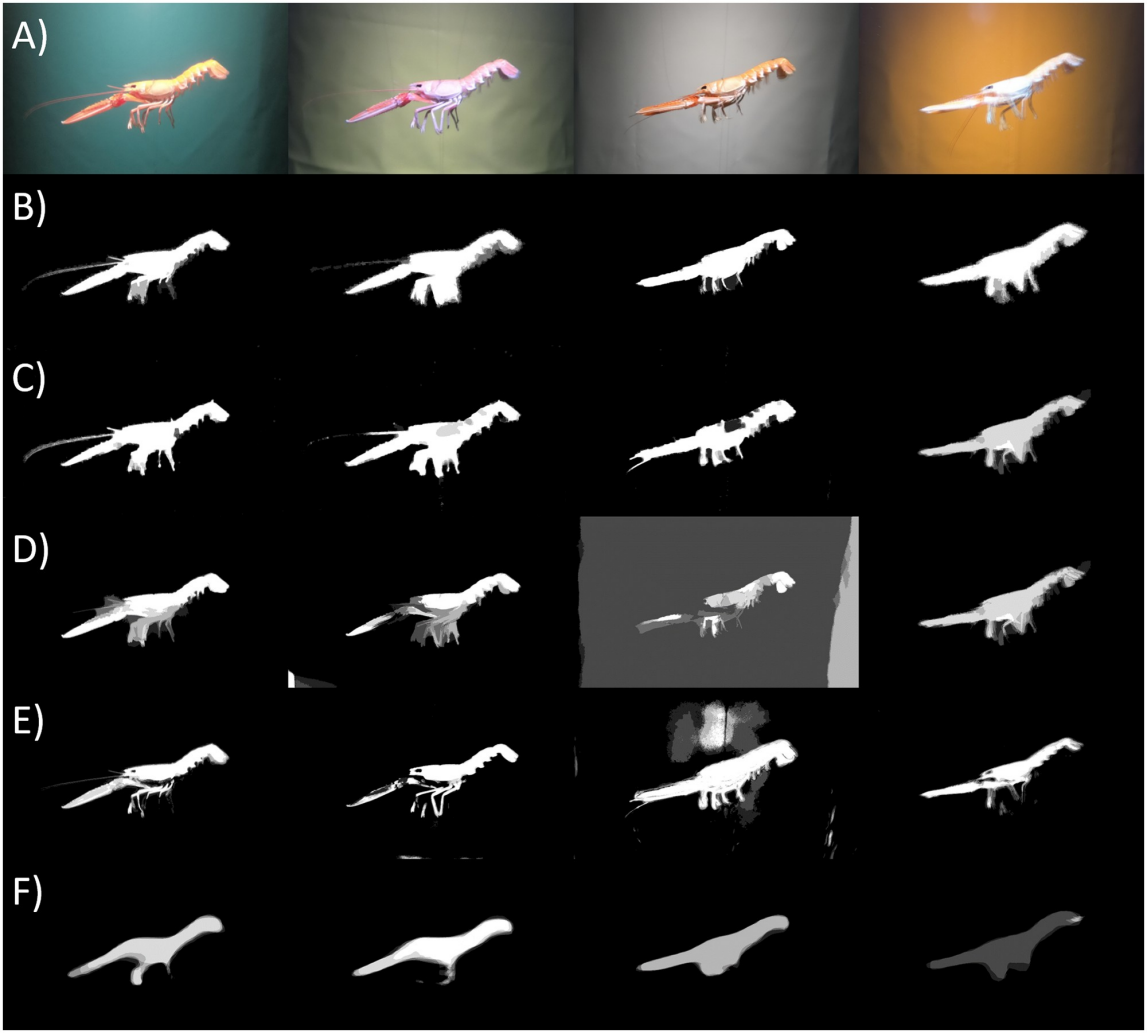
Comparison of the segmentation results. A) Mean images representing the *N*. *norvegicus* fixed in front of the each of the experimental color backgrounds; B) Mean ground truth masks obtained by selecting the regions of interest via Image Labeler MatLab App; C) Predicted object mask based on filling the edge detected by Laplacian of Gaussian; D) Mean predicted masks based on Graph cut segmentation; E) Mean predicted mask obtained by the Random Forest classifier; F) Mean predicted mask by the mask R-CNN pre-trained on the COCO dataset.

The experiment consisted of three parts. The first part included images acquisition of the fixed *N*. *norvegicus* in front of each background (Fig 2A). The experiment was done in two days, therefore two different individuals of the same size were used.

The aim of the second and third parts was to record video of single and multiple *N*. *norvegicus*, respectively, passing with the water current in front of the camera. Videos were sampled at 30 frames per second, 1440p (4:3) resolution, the rest of the settings were set to default.

### Data analysis

#### Image datasets preparation

**Fig 3:**
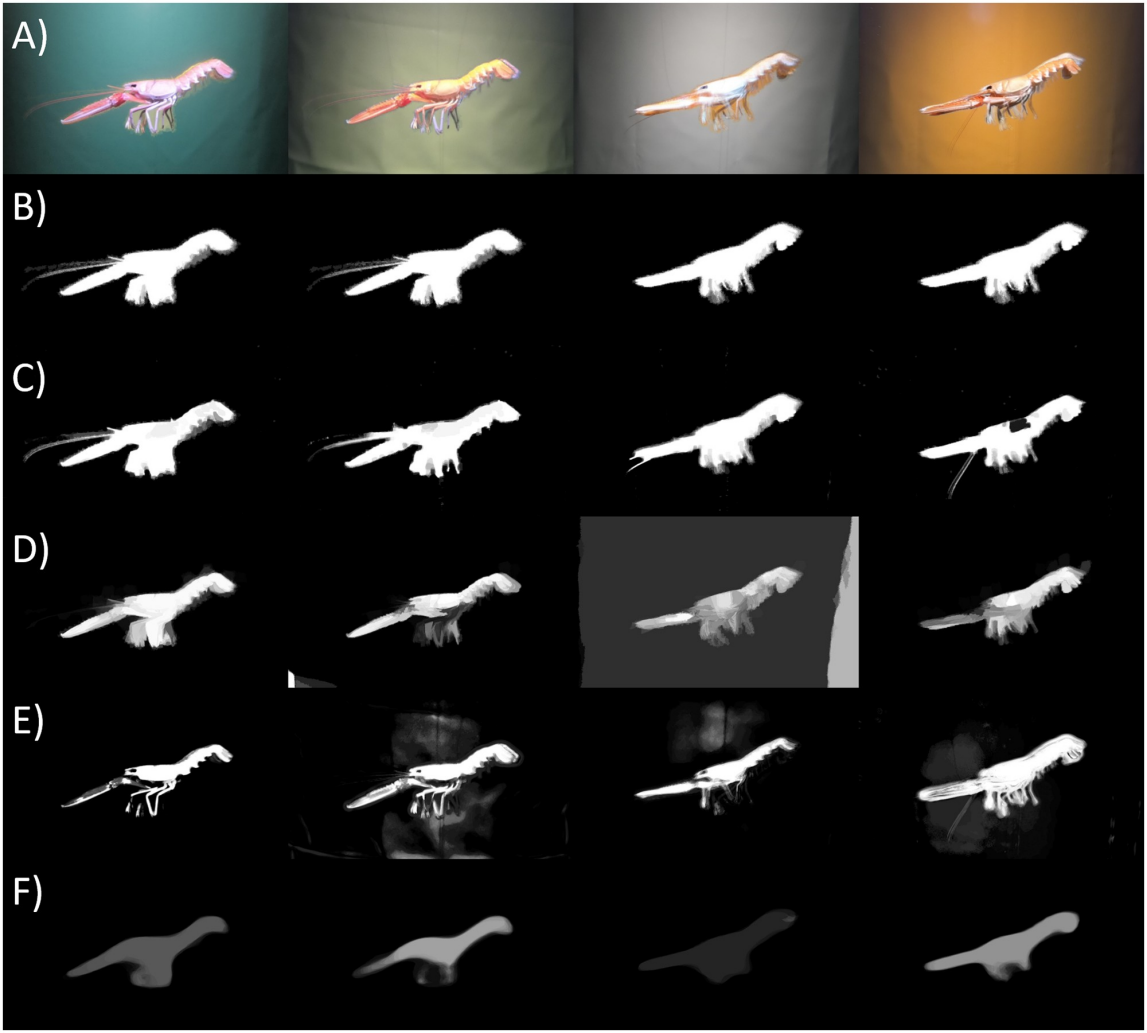
The augmented images. From left to right: A) the mean yellow foreground on the mean green background (YG); the mean green foreground on the mean yellow background (GY); the mean orange foreground on the mean white background (OW); the mean white foreground on the mean orange background (WO); B) Mean ground truth masks obtained by selecting the regions of interest via Image Labeler MATLAB App; C) Predicted object mask based on filling the edge detected by Laplacian of Gaussian; D) Mean predicted masks based on Graph cut segmentation; E) Mean predicted mask obtained by the Random Forest classifier; F) Mean predicted mask by the mask R-CNN pre-trained on the COCO dataset.

The image set collected for the four different backgrounds consisted of 28 images (7 for each background). To keep only the relevant information of the image and to use only the background color itself (without the effect of the part of the tank visible on the sides of the image) we have cropped the original images with keeping *N*. *norvegicus* in the middle and cropping the image down to 2500 pixels in width by 1500 pixels in height. To check the effect of the different foreground appearance on the segmentation output from the background color we have used the simple copy paste augmentation techniques to substitute the original foreground with a foreground taken from another color set without applying any geometric transformations (Fig 3A) [29].

To test the accuracy of the object segmentation we have manually created the binary ground truth masks for each of the image in the dataset using the MATLAB^®^ Image Labeler app polygon ROI assisted labeling assisted freehand method (Fig 2B). To create the ground truth mask for the augmented images we used binary OR operation on the original ground truth masks of the both images from which the foreground and the background were used (Fig 3B).

### Object segmentation

To check whether the specific background results in a better segmentation of the target object we have compared the predicted masks for images in the four datasets with alternative models based on: edge detection, Graph cut, Random forest classifier, pre-trained mask R-CNN model. The scikit-image library [30] was used to implement the Graph cut and Random forest classifier segmentors.

The accuracy of the segmentation prediction was estimated using the Jaccard index (JI) [31] of similarity calculation computing the intersection of the binary images of the predicted binary mask (X) against the ground truth mask (Y) (Eq 1):

$$J=\frac{\left|X\cap Y\right|}{\left|X\cup Y\right|}$$ (1)

Edges in an image are significant local changes in pixel intensities associated with the boundary of an object in the scene [32]. Edge detection is based on isolating local maxima of gradient magnitude by non-maximum suppression along the local gradient direction by Laplacian of Gaussian kernel. It uses zero crossings of second derivatives for precise edges localization [18].

Graph cut segmentation approach treats each pixel as a node in a graph to combine them in superpixels based on the similarity between neighboring pixels. In the method we used the superpixels are determined according to the Simple Linear Iterative Clustering (SLIC) algorithm, which defines the superpixels based on the color and spatial proximity of the candidate regions [33]. Region adjacency graph provides a simple-connectivity view based on the mean color [34]. Dividing the foreground and the background is then done by a normalized cut [35].

Random forest is an ensemble model consisting of multiple decision trees [36]. After multiple trees are generated, in this study the number of trees was set to 50, they vote for either the foreground or the background class of each pixel. As we test the effect of the background color on the segmentation we use pixel intensity and texture features as inputs to the decision trees. Random forest predicts the two classes based on the small training examples, which are the marked groups of pixels in the image. The foreground label, 100 by100 pixels centered on the object and the first 100 rows from top were used as the labels for the background. The Random forest classifier was applied via scikit-learn module in Python 3.7 [37].

Mask R-CNN is an extension of the faster R-CNN with the extra to the existing in faster R-CNN branch for bounding box recognition, where mask runs in parallel to it. Mask R-CNN is an instance segmentation technique that allows to predict not only the bounding box but also the pixel region of an object [22].

To further estimate the effect of the different background colors we used the mask R-CNN trained on the Common Objects in COntext (COCO) dataset [38] to predict the regions and masks for *N*. *norvegicus*. Each of the image of the four backgrounds was used to predict the mask. The model was set to run in inference mode to only predict the mask for the image segmentation task and to predict the mask and the bounding box in the videos; the class prediction was omitted. The key steps of each of the algorithms are presented in Table 1.

**Table 1:** Steps for each segmentation algorithm.

Method	Input	Step 1	Step 2	Step 3	Step 4	Step 5	Output
Edge Detection	RGB image	Convert to gray scale	Image smoothing	Edge detection (LoG)	Morphological closing and flood-fill		Binary mask
Graph cut		Convert to CIE-Lab	Equalize L-channel	SLIC superpixel segmentation	RAG Construction using mean color	Normalized Cut into FG/BG clusters	
Random forest		Convert to HSV	CLAHE on Value channel	Training labels of the FG and BG	Train a Random forest classifier	Predict the FG and BG	
Mask R-CNN		The mask R-CNN implementation follows implementation on Keras and Tensorflow by Matterport [39]				Merge (binary OR) foreground masks	

### Automated *N*. *norvegicus* detection, tracking and counting

The videos were processed with the aid of computer vision toolbox in MATLAB^®^ 2019a. In the computer vision based approach, we combined three foreground detection methods: background modelling and subtraction, red channel thresholding, and low-contrast histogram equalization thresholding (S4 Fig). The first branch of the detector is based on the background subtraction model using Gaussian Mixture Models (GMM) [40]. Compared to the segmentation methods used for background evaluation, this method considers temporal change in video frames and a GMM of the background. In this study, we used 5 Gaussians, 140 training frames and a 0.64 minimum background ratio. We then performed opening and closing morphological operations on the output of the GMM to remove noise, a square structural element was used of size 3 for opening and 15 for closing [41].

The key feature of the remaining two filters is based on the enhanced contrast of the target object and utilizes the red channel thresholding to ultimately separate *N*. *norvegicus* from the background. Thus, the second branch is a red channel thresholding filter (middle branch in S4 Fig), which is used to pick up red components in the image (such as *N*. *norvegicus*). To reduce the influence of varying light conditions in the red channel we subtract the gray scale frame from the red channel frame. We then apply a very low intensity threshold on the red channel intensity of 1%.

The mean intensity shifted histogram equalization filter (right branch in S4 Fig) aims to first reduce the image contrast by subtracting the mean intensity image (across all channels for each pixel), and clipping the intensities outside the interval [0, 1], then restore contrast the image through histogram equalization (CLAHE). The mean intensity shift reduces or removes low intensity color components, resulting in pixels that are dominated by either red, green, or blue colors. Histogram equalization then balances the intensity of the pixels so that contrast is restored [42]. The red channel is then extracted, and a low threshold is applied to remove low intensity noise artifacts from the resulting mask.

The three resulting masks multiplied elementwise (multi-AND operation) which is then passed to the blob analysis function. The blob analysis function, provided in MATLAB^®^, identifies groups of connected foreground pixels (blobs) that are separated from each other and within a specified size (minimum blob area is set to 7500 pixels). It then provides bounding box position and size information for each detected blob.

As a second object-detection method, the mask R-CNN architecture with weights pre-trained on the COCO dataset (as used in background evaluation) was selected. As the initial network was trained on 80 classes of objects and we are controlling that only *N*. *norvegicus* objects pass the camera, we can simply treat all detected classes as the same instance class. Thus, in our setup mask R-CNN is not used for object classification, we just use the bounding box position and size information from each detected instance.

The tracking part of the algorithm (S5 Fig) was the same and, in both cases, the bounding box information was used as input. The object tracker consists of an object-to-track assignment component and a Kalman filter-based track prediction component [43]. Each object track is considered unique and are tallied for automatic counting.

The objects are assumed to be moving with a constant velocity through the image, so the Kalman filter model is first order (second derivatives are set to 0). This assumption reflects our use case: *N*. *norvegicus* are expected to tumble through the trawl net at a near-constant speed and are unable to swim with the trawl [44]. Detected centroids that are assigned to tracks are then used in the update step of the assigned Kalman filter. Tracks that have not been updated for several consecutive frames are then marked as lost (indicating it is likely that the object has left the image). Detected objects that have not been assigned to a track by the assignment component are initialized with a new Kalman filter tracker. Each unique track is counted and contributes to the automatic count of *N*. *norvegicus* from the input video.

The assignment component uses the Hungarian algorithm [45] to match observed object centroids for the current frame with predicted track centroids for any existing tracks. The track prediction component is a Kalman filter, initialized upon the detection of a new object that predicts the position of the assigned object. A cost matrix for the Hungarian algorithm is constructed as the matrix of Euclidean distances between detected object positions and predicted positions of tracked objects.

A total of five video files were used for evaluating the algorithms: three with single individuals dragged across the camera’s field of view and two videos containing several *N*. *norvegicus* freely passing in front of the camera with the water flow. The evaluation was based on comparing the automatic count with a manual count made by a human watching the video. Furthermore, the human supervisor counted the number of false positives (times when the algorithm identified an object when there was none) and false negatives (times when the algorithm missed an object).

## Results

### Object segmentation

Segmentation applied to the original images showed the highest JI values for the green background images set (JI = 0.72). *N*. *norvegicus* on white and orange backgrounds was segmented from the background with the average JI = 0.5. *N*. *norvegicus* on the yellow background provided the second segmentation results (JI = 0.63).

Among the four segmentation techniques the edge detection segmentation provided the highest results for all color sets and on average JI equaled to 0.75 (Table 2). The mean predicted masks by each of the techniques is presented in Fig 2C–2F.

**Table 2:** The mean Jaccard index (JI) scores for the four segmentation techniques applied to the original images.

Segmentation	Green	Yellow	White	Orange	Average JI per segmentation
LoG edge detection **±** STDEV	0.80±0.04	0.79±0.04	0.76±0.04	0.66±0.27	0.75±0.1
Graph cut **±** STDEV	0.76±0.06	0.59±0.07	0.22±0.19	0.56±0.2	0.53±0.13
Random forest **±** STDEV	0.71±0.12	0.55±0.07	0.48±0.07	0.57±0.06	0.58±0.08
Mask R-CNN **±** STDEV	0.61±0.27	0.58±0.08	0.55±0.38	0.22±0.36	0.49±0.27
Average JI for the color set	0.72±0.12	0.63±0.07	0.5±0.17	0.5±0.22	

The presented values of Jaccard index are the means of the individual indexes obtained for the individual image ± Standard Deviation (STDEV). The original set contained seven images for the each of the four test background colors.

In case of augmented images the segmentation based on the edge detection provided predicted masks of high accuracy for all four sets (0.77 < JI < 0.82) (Table 3). The Graph cut method of the foreground-background separation based on the mean color showed the best segmentation results for the green background among the four combinations: JI = 0.77. The Random forest classifier showed better segmentation results for the white background JI = 0.67, whereas the JI-values for the other three background colors were very similar and ranged between 0.46 and 0.48. The foreground mask prediction obtained by the pre-trained mask R-CNN increased among the four background colors from JI = 0.12 to JI = 0.42 by on average 0.1 for the orange, green, yellow and white backgrounds respectively. The mean predicted masks by each of the techniques are presented in Fig 3C–3F.

**Table 3:** The mean Jaccard index (JI) scores for the four segmentation techniques applied to the augmented images.

Segmentation	YG	GY	OW	WO	Average JI per segmentation
LoG edge detection **±** STDEV	0.80±0.05	0.78±0.04	0.77±0.03	0.82±0.02	0.79±0.04
Graph cut **±** STDEV	0.77±0.12	0.53±0.1	0.62±0.19	0.35±0.16	0.57±0.14
Random forest **±** STDEV	0.48±0.08	0.48±0.1	0.67±0.06	0.46±0.16	0.52±0.1
Mask R-CNN **±** STDEV	0.26±0.36	0.35±0.28	0.42±0.29	0.12±0.37	0.29±0.36
Average JI for the color set	0.58±0.15	0.53±0.13	0.62±0.14	0.44±0.27	

The presented values of Jaccard index are the means of the individual indexes obtained for the individual image ± Standard deviation (STDEV). The augmented images set consists of the images with the foreground taken from the other color set: the letters in abbreviation correspond to the color first letter from where the foreground and the background, respectively was used for making the augmented images.

### Automated *N*. *norvegicus* detection, tracking and counting

Application of the proposed computer vision (CV) detector followed by motion-based tracking algorithm on the video dataset showed high *N*. *norvegicus* detection, tracking and counting accuracy (Table 4). The algorithm performed better for the single *N*. *norvegicus* passing video types, with lower accuracy for the multiple *N*. *norvegicus* videos.

**Table 4:** Results of the automated detection and count of *N*. *norvegicus*.

Single (S)/Multiple (M) targets	Video length (seconds)	Manual count	Total automatic count (CV | MR-CNN)	True positive (CV | MR-CNN)	False positive (CV | MR-CNN)	False negative (CV | MR-CNN)	Precision (CV | MR-CNN)	Recall (CV | MR-CNN)	F-score (CV | MR-CNN)
S 1	283	40	40 | 139	37 | 36	3 | 103	3 | 4	0.93 | 0.26	0.93 | 0.90	0.93 | 0.20
S 2	136	19	17 | 73	16 | 17	1 | 56	3 | 2	0.94 | 0.23	0.84 | 0.89	0.89 | 0.18
S 3	403	74	65 | 239	64 | 69	1 | 170	10 | 5	0.98 | 0.29	0.86 | 0.93	0.92 | 0.22
M 1	146	11	14 | 24	6 | 8	8 | 16	5 | 3	0.43 | 0.33	0.55 | 0.73	0.48 | 0.23
M 2	152	7	13 | 18	6 | 5	7 | 13	1 | 2	0.46 | 0.28	0.86 | 0.71	0.60 | 0.20

CV, the proposed computer vision algorithm and MR-CNN, the pre-trained mask R-CNN detection followed by the Kalman filter applied on the videos of the single (S) individual dragged in front of camera, whereas a multiple (M) target set was a group of *N*. *norvegicus* released in the tank.

**Fig 4:**
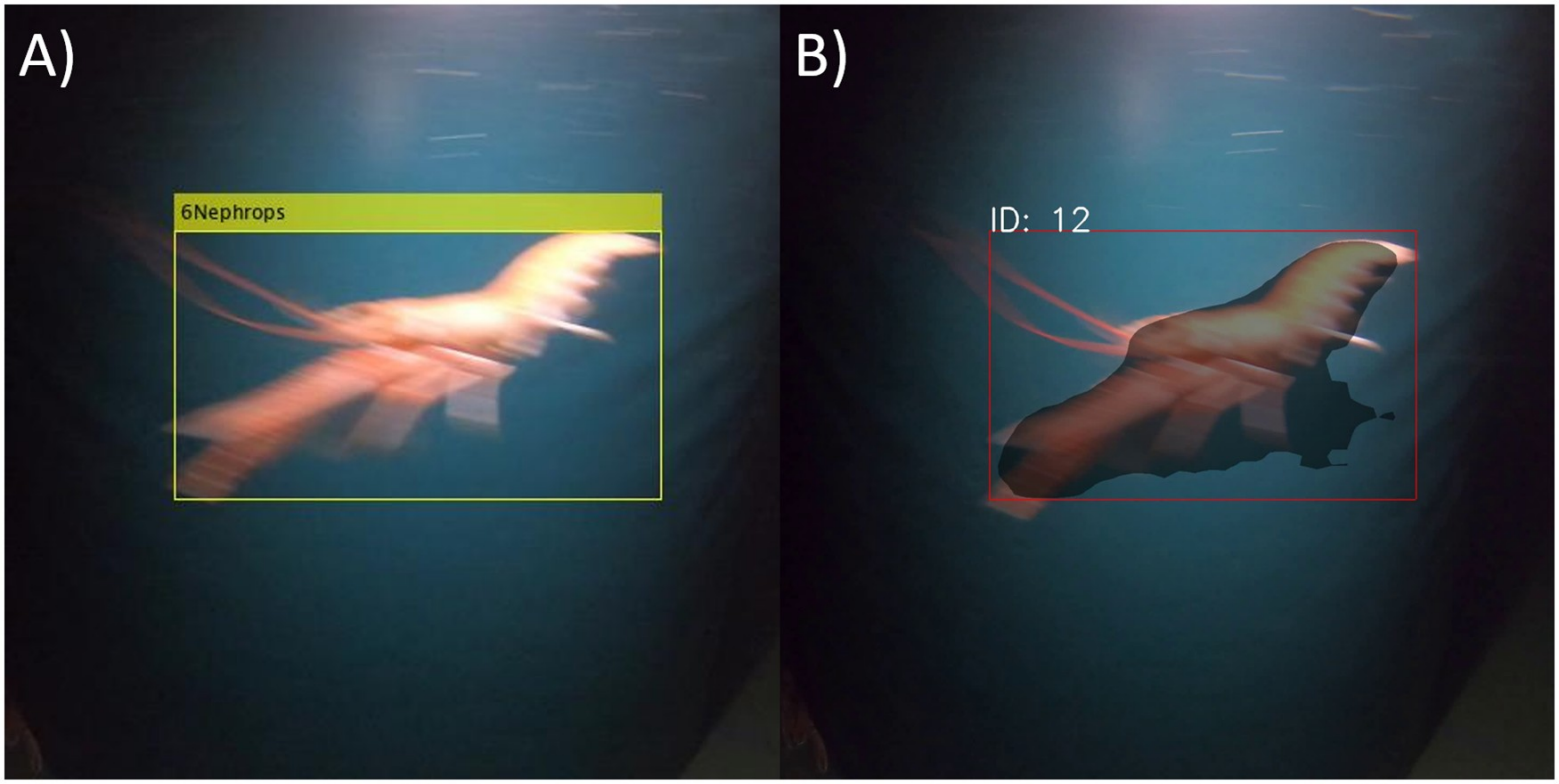
Example of detected and tracked *N*. *norvegicus*. (A) The output of the proposed computer vision algorithm; (B) the output of mask R-CNN pre-trained on COCO.

Fig 4 provides an example of successful detection and assigned tracking number to the *N*. *norvegicus* from both the blob analyzer (A) and mask R-CNN (B).

To better understand the performance of the algorithm, we have counted the number of false positive and false negative detections. False positives occur when noise was detected as *N*. *norvegicus* or when a single *N*. *norvegicus* was detected as multiple individuals. False negatives occur when *N*. *norvegicus* was present in the frame, but missed by the algorithm. These values were used to estimate the precision, recall and F-score performance of the algorithm.

Like accuracy, these three metrics were larger (indicating better performance) for the single target compared to multi-target videos.

For the single target videos, the precision was higher than the recall, whereas for the multiple objects the recall was higher comparing to the precision (Table 4).

The pre-trained mask R-CNN detector, used instead of the blob analyzer showed a higher number of false positives that was also indicated by lower precision. However, the number of the false negatives was comparable with the blob detector (Table 4).

### Real-time performance assessment

We have recorded the time taken for the classic computer vision algorithm to process, view the processed video frames as they are completed and save the results in a new video file. The test videos were processed using an Intel^®^ Core^™^ i5-8250U CPU 1.60GHz processor with 16 GB RAM. Given these settings and hardware the algorithm processes on average one frame per 2.5 frames of the input video stream.

Assessing only the algorithm (without displaying or saving the processed video just providing the count) it processes one frame per 1.5 frames of the input video. With further optimization of the code and utilization of parallel CPU cores the performance of the algorithm is expected to become close to or even exceed one processed frame per video input frame.

To run the algorithm with mask R-CNN as the object detector we used an Intel^®^ Core^™^ i7-8750H CPU 2.20GHz processor with a 16 GB RAM, NVIDIA Quadro P1000 Graphic card with 640 NVIDIA CUDA^®^ Cores and 4 GB GPU memory. Given these settings and hardware the algorithm processes on average one frame per 31.5 frames of the input video stream.

## Discussion

In this study, we presented an image acquisition system (NepCon) for *N*. *norvegicus*-directed trawl fishing, and evaluated the effect of the high-contrast background on the object segmentation and automatic *N*. *norvegicus* detection, tracking and counting based on computer vision and deep learning algorithms. In this first phase NepCon was developed under fixed experimental conditions with the controlled light source as well the opaque contrasting material used as a background.

### Background color choice

Several factors contributed to the selection of background color and material: color visibility under water, custom color hue, segmentation results, potential visibility for the fishes (for at-sea implementation and the effect on fish behavior), camera automatic settings response to the particular color.

The background color influences the object appearance in case the automatic camera settings are chosen (Fig 2A and S1 and S2 Figs). The shift in object hues may preserve the efficiency of object segmentation, however it complicates the evaluation of the object classification. Yet, the use of the camera automatic settings allows the system to be more adjustable for the possible variations in lightning, such as back-scattering of the light from the reflective fish scales and the sediment.

Orange is the most visible color in turbid waters and *N*. *norvegicus* were distinguishable on this background. However, the hues of *N*. *norvegicus* were shifted more towards blue, and additionally orange background could possibly yield in higher visibility for fish in trawl.

White is a uniform color, and when used as a background it makes all items visible with the camera settings reflecting the ‘true’ color of the object. However, most fishes and aquatic organisms contain white color in their pigmentation that may affect the object detection accuracy and potentially the size estimation of the object due to less accurate detection in case of partial detection. A white background is expected to make the dark sediment more visible leading to poorer image quality and higher chance of the false positive detections.

Green is a complementary color to red (the color of the target species) [18, 27], and it creates a good contrast yielding in better segmentation results. *N*. *norvegicus*, like several other species of crustaceans, reflect red-band wavelength light due to the presence of carotenoid pigment, astaxanthin, in the carapace [46]. The sediment and algae are expected to be less visible on the darker green background due to similar wavelength of the reflected light.

Yellow is the second most visible color in turbid waters after orange and showed the second segmentation results for *N*. *norvegicus* according to Jaccard index (Table 2). We expect the higher sediment visibility on this brighter than green background.

### *N*. *norvegicus* segmentation

We have evaluated the accuracy of the segmentation techniques by calculating the Jaccard index that compares the similarity between the predicted object mask with the ground truth mask. The ground truth has been generated using the pixel label assisted freehand that helps to create the more accurate object mask with minimum presence of the background pixels in the foreground mask. However, with this technique the background pixels are not entirely eliminated from the foreground mask hence resulting in a bias in the Jaccard index values.

The segmentation techniques applied on the augmented data showed higher variability in the results (Table 3). Due to the ground truth labeling process, the foregrounds pasted onto different backgrounds contain small amounts of pixels belonging to the original background (Fig 3A and 3B), this biases the segmentation results, however all the augmented images have the same source of bias with the same relative contamination. The Graph cut segmentation method showed the highest results for the green background (Table 3). As this technique is based on mean color distance for the foreground-background separation, it supports the hypothesis that the better color contrast provides better segmentation results at least for color-based techniques. Here we can see that the shift in the foreground hue still provides the best segmentation output. The obtained result correlates with the larger distance between the foreground and the background of the tested colors (S3 Fig).

### *N*. *norvegicus* detection, tracking and counting

In a stable observation scene (i.e. controlled illumination and an opaque background of a specific color), the proposed algorithm based on the blob detector can detect, track and count *N*. *norvegicus* with an F-score of 76.4%. The accuracy of the algorithm was higher for the videos containing individual *N*. *norvegicus* compared to the accuracy for the multiple individuals passing. The observed result can be explained by the fact that the algorithm tends to inaccurately separate the individuals when they are occluding each other while formed in groups. Blob detector is semi semantic, in that it attempts to identify instances within a binary image, whereas mask R-CNN creates a binary image for each instance. Besides this, some individuals are being detected in parts, i.e. parts of a single *N*. *norvegicus* are being identified as separate *N*. *norvegicus* entities. These issues may be solved by proceeding with advanced feature-based segmentation methods. The deep learning methods show promise as they consider a wider range of learned object features in addition to color and area of interest size however they require a large amount of the training data [20, 47]. In this study, we have used the pre-trained mask R-CNN as a substitute of the blob analyzer. Even without performing the transfer learning of the model on the collected data, the network is able to accurately predict regions of interest, however the mask R-CNN detector resulted in a higher number of false positive detections in both single and multiple target videos. This method has a large potential to outperform the proposed blob analyzer object detection and to be more robust in terms of low sensitivity to other objects and noise that can be present in the frame during fishing [19].

Mask R-CNN has already found an application in solving different tasks related to fishery. For instance, it has been used for automatic fish species detection and classification in the images collected during the scientific monitoring [23, 48]. It has also been used in assistance to the analysis of videos obtained during electronic monitoring onboard the fishing vessel [49]. The pixel-wise mask output of the model helped to estimate the total length of the wild and farmed harvested fish [24, 25].

The multi-object tracking component of the algorithm does not provide an issue in terms of accuracy, however the object detection component is where most of the inaccuracy can be attributed. This is due to incorrect detection (false positive) and missed target object (false negative) errors. The occurrence of the false positive and false negative detections can be explained by the general approach that is used in the filtering masks, blob analyzer and the input data quality which is dependent on the sampling conditions.

The application of the deep learning detector in its current configuration is however not applicable for near real-time performance, whereas the blob analyzer is closer to real-time and, under the controlled conditions, is able to provide an accurate automated catch indication.

### Applications of NepCon

The NepCon system can be used not only in fisheries, but also have an application in the monitoring of aquatic resources. For instance, the automated recognition and removal system of the Asian carp [50] can benefit from the contrast enhancement used in NepCon and detect and remove species based on more basic object features instead of applying the genetic algorithm.

Fish, both fresh water and marine, are the most common target for automatic detection for the fishing industry. The approaches aim to detect targets both *in situ* underwater and on deck after fishing is over, for example, on the conveyer belt during sorting, to estimate the amount of bycatch or to assist the manual catch sorting [51]. The object recognition on the conveyer belt is easier than underwater since it is performed with a stable, highly controllable background with lighting, whereas underwater conditions are much more challenging, especially close to the seabed where mobilized sediment can occlude the image. Thus, the presented automated algorithms used for *N*. *norvegicus* detection and count may be applied on the on-deck catch monitoring.

Computer vision found an application in a study of *N*. *norvegicus* behavior [52]. To distinguish between individuals and identify their movements the authors designed tags that were attached to the animals’ cephalothoraxes and were used as references for automatic detection. The NepCon can be applied here without the need to design and attach tags to animals, instead *N*. *norvegicus* can be automatically detected either fully or partially and tracked.

In-trawl catch monitoring systems are being tested in pelagic research trawls to verify the catch species composition captured by the echosounders [13]. The data obtained by such a system can also be processed automatically [23]. The system may benefit from the NepCon concept by applying the contrasting background to the target species which enables the computer vision based object recognition techniques and improves the performance of the deep learning model for the object segmentation task.

## Conclusions and future work

We have developed and demonstrated a design concept for underwater image acquisition of *N*. *norvegicus* using opaque contrasting materials as a background and further explored the possibility of automatic detection and counting of one of the most challenged and economically valuable species in the European demersal trawl fisheries.

Such system can be implemented by both fishers, to monitor ongoing catch rates, and by researchers performing monitoring operations. The technology can work as an assistant to fishers by using the core algorithm to provide a catch estimate, besides, the algorithm can be used for selecting frames of interest for the deep learning algorithm training.

Our study presents a novel approach to the demersal fisheries field by introducing a stable, robust and cost-effective image acquisition system for underwater environments, specifically tuned for automatic detection and tracking of *N*. *norvegicus*. This experimental development and test of concept under controlled conditions is an important step before transferring the system to real fishing conditions and before expanding the database for further improvement of the automatic detector. The future in-trawl system integration should maintain consistent light conditions as well as an opaque high-contrasting background. Using tarpaulin, or similar material as a background instead of colored netting will minimize influence from external variation in light and seabed color that can be visible through netting. Despite the optimization of the observation scene setup the mobilized by the ground gear sediment may to some extend challenge the quality of the image acquired during the demersal trawling.

Semantic and instance segmentation methods, which have been achieved through several powerful deep learning approaches, could improve upon the proposed solution provided enough training data is collected.

Therefore, future work includes integrating an observation section inside the demersal trawl gear, containing a fixed green background with sufficient artificial light and a camera overviewing the passing catch elements as the main components. When operational in trawl, extensive images for the application of state-of-the-art deep learning segmentation algorithms should be collected.

## Supporting information

S1 Fig Comparison of the gray scale mean images histograms: A) rgb2gray (luminance) Chi-squared distances between the histograms of the four test backgrounds; B) rgb2lightness (lightness) Chi-squared distances between the histograms of the four test backgrounds; C) rgb2gray (luminance) Euclidean distances between the histograms of the four test backgrounds; D) rgb2lightness (lightness) Euclidean distances between the histograms of the four test backgrounds. (TIF)

S2 Fig Comparison of the gray scale mean foreground (FG) histograms: A) rgb2gray (luminance) Chi-squared distances between the histograms of the four test backgrounds; B) rgb2lightness (lightness) Chi-squared distances between the histograms of the four test backgrounds; C) rgb2gray (luminance) Euclidean distances between the histograms of the four test backgrounds; D) rgb2lightness (lightness) Euclidean distances between the histograms of the four test backgrounds. (TIF)

S3 Fig Comparison of the foreground (FG) and background (BG) in the four mean images: A) Euclidean distance B) Mahalanobis distance. (TIF)

S4 Fig Flowchart indicating steps of the object detection part of algorithm based on the computer vision approach. The left branch indicates the foreground detector filter using Gaussian Mixture Models (GMM); the middle branch indicates thresholding on just the red channel of the frame. The right branch indicates the mean intensity shift and histogram equalization filter.
(TIF)

S5 Fig Flowchart indicating steps of the automated object tracking and counting. (TIF)
