# Supplementary figures and images for: Towards sustainable demersal fisheries: NepCon image acquisition system for automatic Nephrops norvegicus detection
Source: PLoS One. 2021 Jun 16;16(6):e0252824. doi: 10.1371/journal.pone.0252824 (PMC8208558; doi:10.1371/journal.pone.0252824)

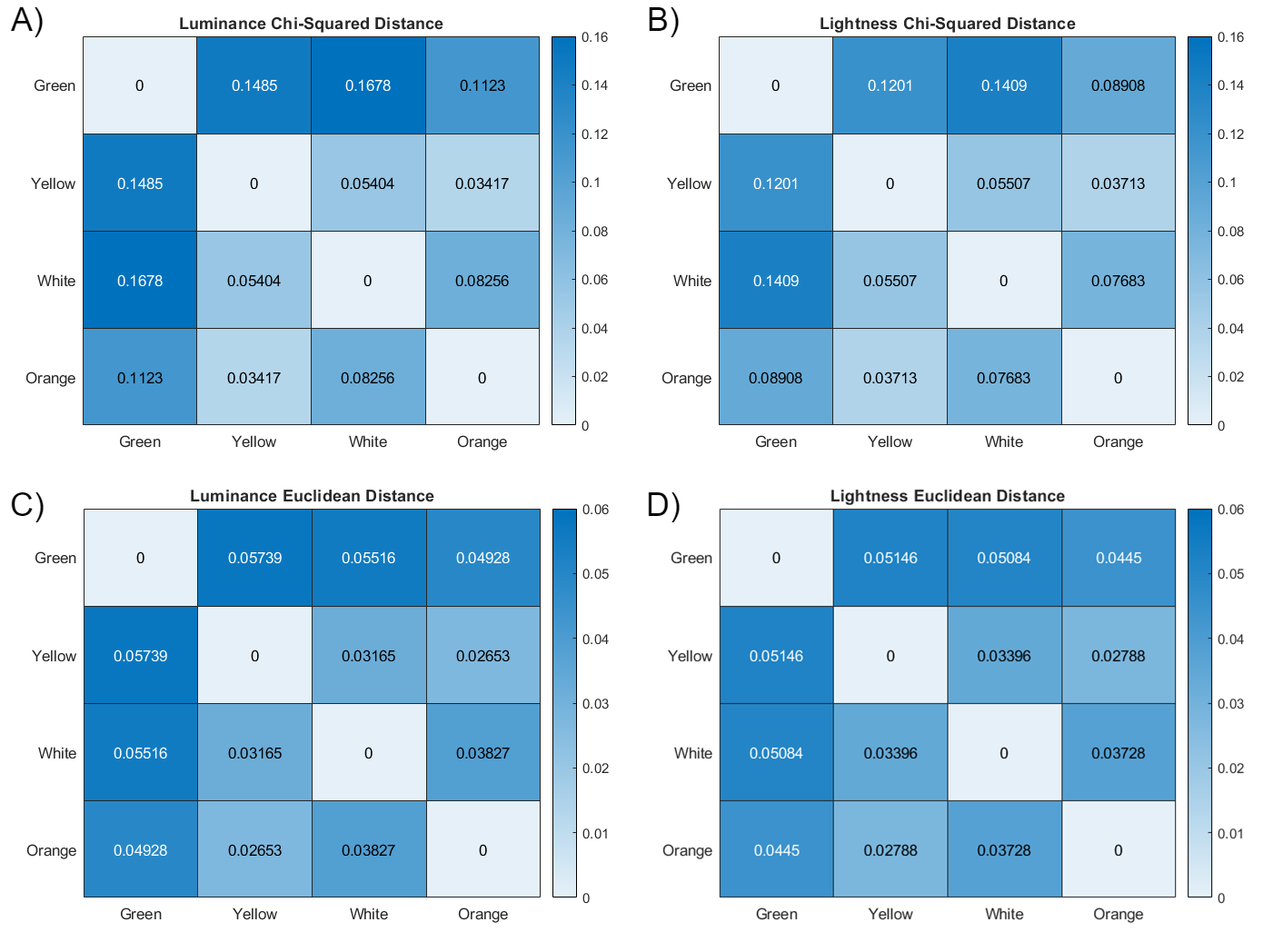

Supplement: S1 Fig — (TIF) [file pone.0252824.s001.tif]

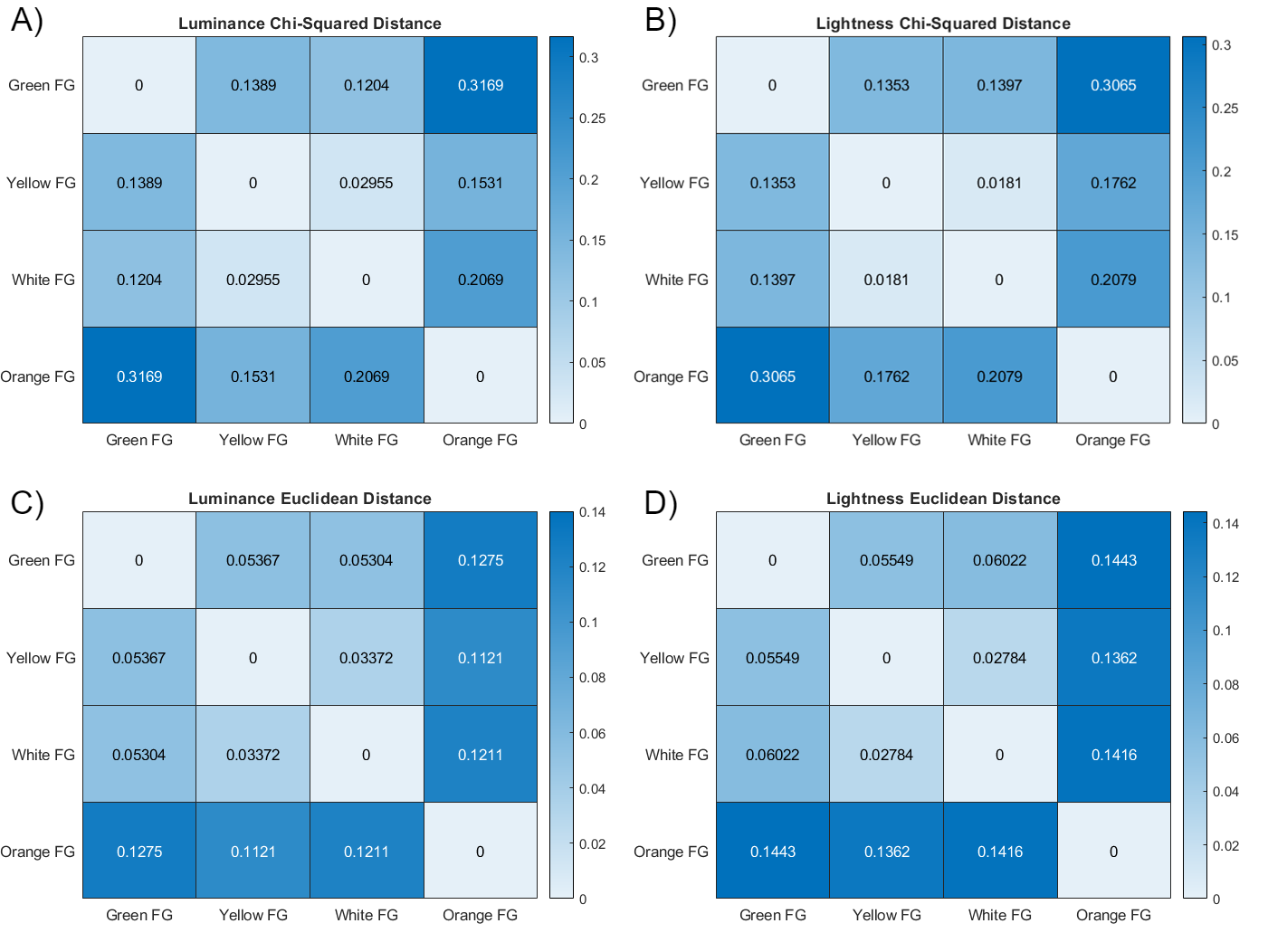

Supplement: S2 Fig — (TIF) [file pone.0252824.s002.tif]

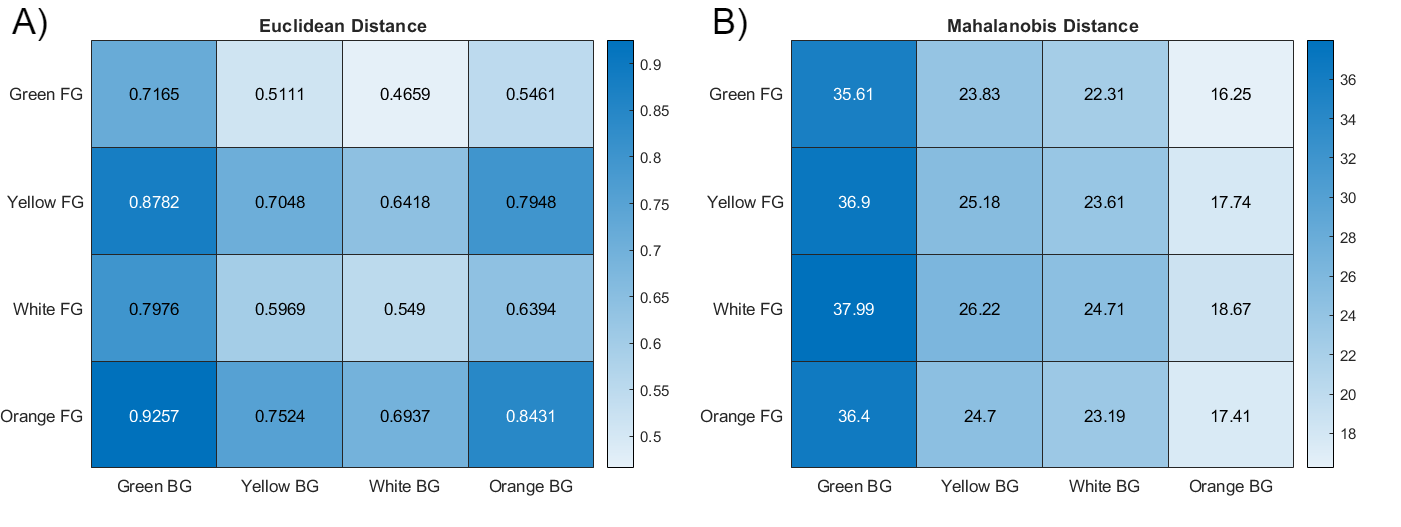

Supplement: S3 Fig — (TIF) [file pone.0252824.s003.tif]

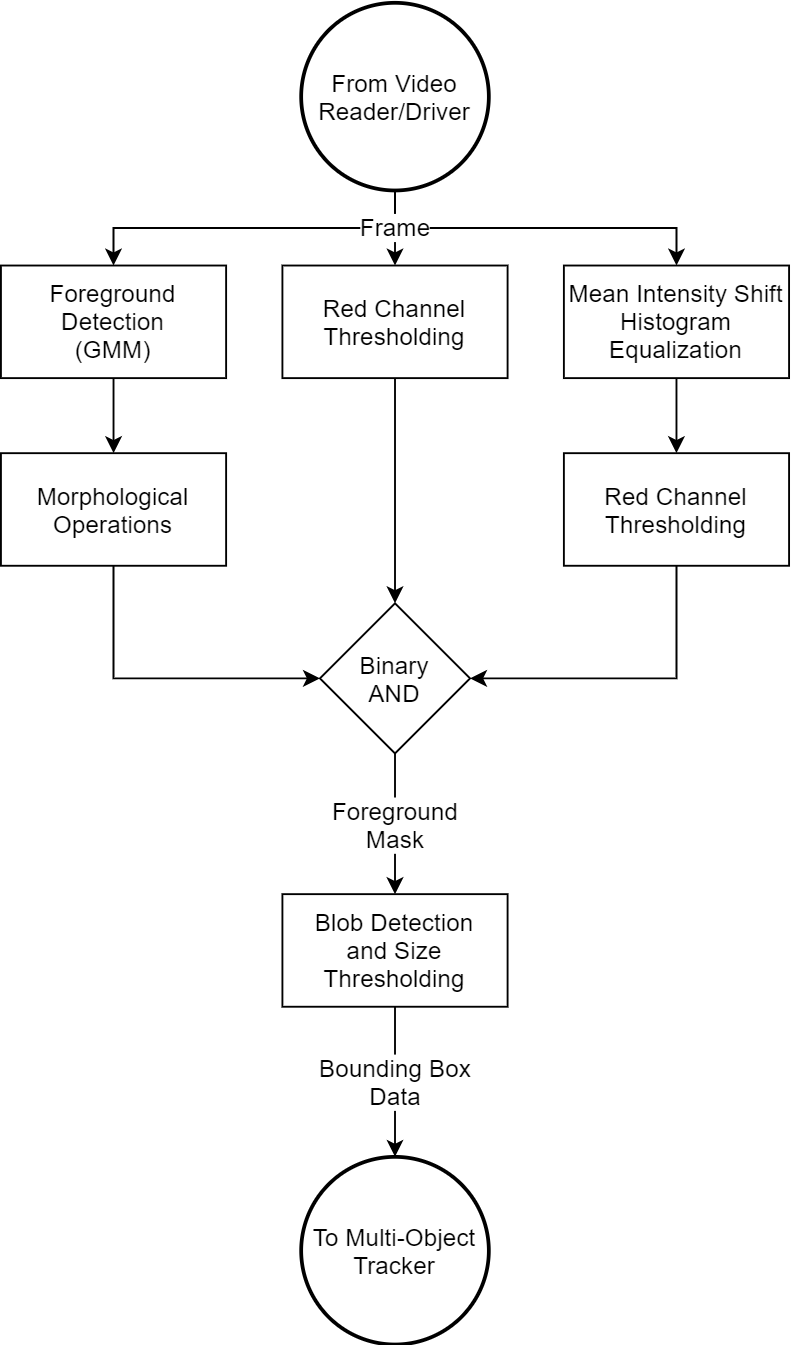

Supplement: S4 Fig — The left branch indicates the foreground detector filter using Gaussian Mixture Models (GMM); the middle branch indicates thresholding on just the red channel of the frame. The right branch indicates the mean intensity shift and histogram equalization filter. (TIF) [file pone.0252824.s004.tif]

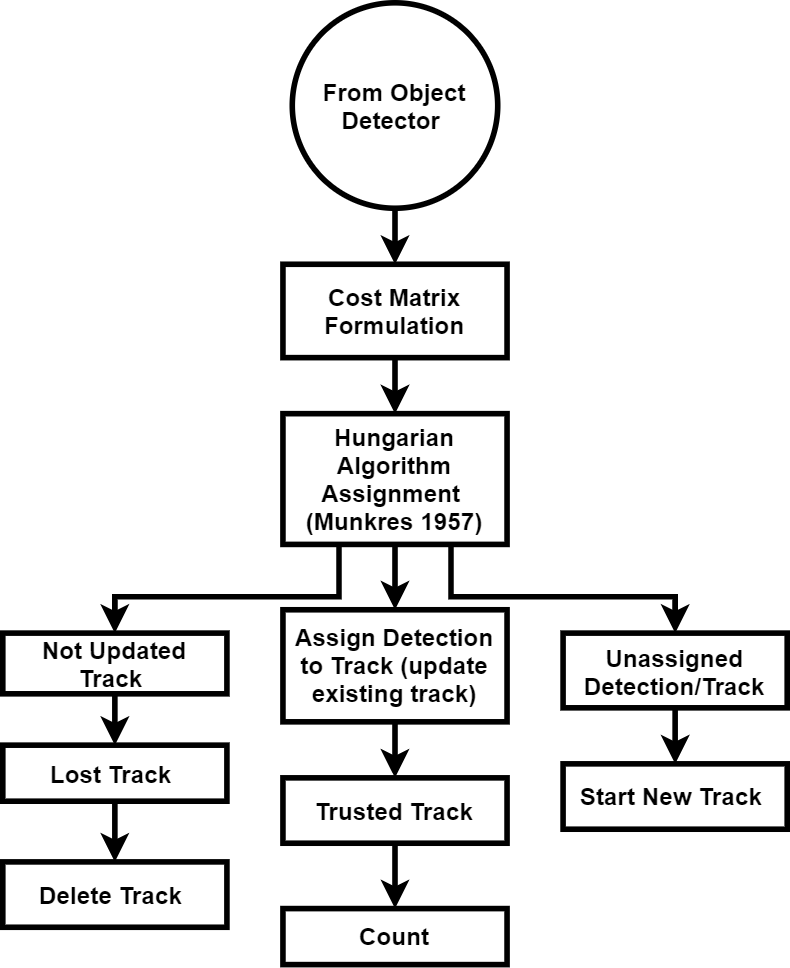

Supplement: S5 Fig — (TIF) [file pone.0252824.s005.tif]
